# The Intraocular Lens Tilt and Decentration of Two Different Hydrophobic Aspheric Monofocal Intraocular Lenses

**DOI:** 10.22336/rjo.2025.57

**Published:** 2025

**Authors:** Yasin Sakir Goker, Serdar Ozates, Mustafa Koc, Halil İbrahim Ateşoğlu

**Affiliations:** 1 Goker Retina and Eye Clinic, Ankara, Turkey; 2 Department Of Ophthalmology, Okan University, Istanbul, Turkey; 3 Ideal Eye Center, Kayseri, Turkey; 4 Tatvan State Hospital, Bitlis, Turkey

**Keywords:** decentration, intraocular lens, phacoemulsification, tilt, visual acuity, IOL = intraocular lens, UCVA = uncorrected visual acuity, BCVA = best corrected visual acuity

## Abstract

**Purpose:**

To evaluate the clinical performance of two monofocal aspheric intraocular lenses (IOLs) to determine their position in the capsular bag after implantation.

**Methods:**

This retrospective study collates data from the medical records of patients who underwent cataract surgery and were implanted with two different hydrophobic aspheric monofocal IOLs. A total of 6 visit data were considered in the study, out of which four follow-up visits were on day 1, 7, 30, and 180 postoperatively. The investigational variables included IOL tilt and decentration, uncorrected and best corrected visual acuity (UCVA and BCVA), contrast sensitivity, manifest refraction, intraocular pressure, and posterior capsule opacification.

**Results:**

A total of 85 subjects’ medical records were screened. Nineteen subjects discontinued the study due to loss to follow-up. Thirty subjects’ medical records were enrolled in the IOL 1 arm, and 36 subjects’ medical records were enrolled in the IOL 2 arm. There were no statistically significant differences in vertical and horizontal IOL tilt and decentration results between IOL 1 arm and IOL 2 arm. Within-group analysis revealed a significant difference in BCVA at all visits from baseline for both the IOL 1 and IOL 2 arms. In the between-group analysis, there were no significant differences in BCVA at any visit between the IOL 1 and IOL 2 arms.

**Discussion:**

Tilt and decentration of IOL may occur secondary to complicated cataract surgery or following uneventful phacoemulsification. IOL tilt up to 2-3 degrees and decentration of 0.2-0.3 mm are common and not clinically significant. Larger amounts of decentration and tilt deteriorate the optical performance of the IOLs, subsequently affecting the patients’ vision.

**Conclusion:**

IOL vertical and horizontal tilt and decentration found in the current study are within the limits reported in the literature. The position of these two lenses in the capsular bag remains very stable at day 180 after implantation.

## Introduction

With technological advancements, cataract surgery has evolved from a purely restorative procedure to one with both restorative and refractive purposes. To achieve a good visual effect after surgery, a thorough evaluation of surgical procedures and the accurate position of an intraocular lens (IOL) are crucial. Improper tilt and decentration of the IOL may lead to deterioration of visual function, characterized by impaired visual acuity and increased higher-order aberrations [1].

The aberrations of the eye depend on the anterior corneal surface, posterior corneal surface, and crystalline lens [2]. The shape and transparency of the crystalline lens are altered by aging, leading to the formation of cataracts [3]. Cataract surgery is planned to improve vision, and the intraocular lenses, which utilize various technologies, enable patients to see better.

Aspherical and spherical IOLs are widely used in cataract surgery. Clinicians often aim to minimize the postoperative aberration of the entire eye and improve the patient’s visual quality. Standard aspheric monofocal IOLs could modify the spherical aberrations of the cornea. These IOLs compensate for corneal spherical aberration, thus reducing the total spherical aberration of the whole eye after cataract surgery and improving visual acuity.

The development of wavefront measurements has raised many still unanswered questions regarding retinal image quality, optical eye performance, neural processing, and the cognitive interpretation of the retinal image, as well as the visual benefits of correcting aberrations [4]. A wide variety of aberrations have been described, and the potential advantages of correcting them have been calculated [1,4,5]. Retinal image quality depends on a complex interaction between optical defocus, astigmatism, and higher-order aberrations (HOAs).

While aiming to correct spherical aberrations, the proper tilt and decentration of IOLs are becoming increasingly crucial [6,7]. IOL decentration has a greater impact on reducing visual acuity than tilt [8]. The axial length also has an essential effect on IOL tilt and decentration after cataract surgery [9].

Concerning routine cataract surgery, aspheric IOLs have been designed to control the amount of total spherical aberrations. Eyecryl Plus ASHFY600 and Optiflex Genesis have negative spherical aberration. Their aspheric optic surface compensates the cornea’s positive spherical aberration. Moreover, these lenses reduce chromatic aberration via their natural yellow chromophore. This chromophore absorbs the UV light and filters violet light.

In this study, the clinical performance of two monofocal aspheric IOLs (Optiflex Genesis (Biotech Europe, Meditech Inc Limited, Ireland) and Eyecryl Plus ASHFY600 IOL (Biotech Vision Care Pvt Ltd, India) was compared to determine their position in the capsular bag after the implantation. The primary objective was to evaluate the IOL tilt and decentration after IOL implantation, and the secondary objective was to assess the visual outcomes and safety of hydrophobic aspheric monofocal intraocular lenses, including the recording of adverse events and serious adverse events.

## Methods

This retrospective cross-sectional comparative study was performed at a tertiary Training and Research Hospital between January 2020 and April 2022. The study was conducted in accordance with the Declaration of Helsinki and was approved by the local Ethics Committee (SBU Ankara Training and Research Hospital Ethics Committee, 12-1-2022, No: E-93471371-514.99).

### Baseline examination protocol and measurements

The investigational variables included IOL tilt and IOL decentration, uncorrected and best corrected visual acuity (UCVA and BCVA) according to the Snellen Chart, contrast sensitivity (measured by Pelli-Robson Contrast Sensitivity Charts) under mesopic conditions monocularly, manifest refraction, intraocular pressure, posterior capsule opacification (PCO), and overall safety with hydrophobic monofocal IOL using treatment-emergent adverse events. The PCO data were graded as none (0 points), mild (1 point), moderate (2 points), or severe (3 points), as assessed by dilated anterior segment imaging in retroillumination on 180 days of follow-up after surgery [10]. Mild PCO was classified as multiple discrete epithelial pearls, moderate PCO was classified as multiple coalescent epithelial pearls, and severe PCO was classified as a thick sheet of epithelial pearls [10].

### Eligibility criteria

Inclusion criteria were age ≥18 and ≤80, best corrected visual acuity of 0.2 LogMar or lower preoperatively, cataract, patients who have already undergone cataract surgery, and Eyecryl ASHFY600 or Optiflex Genesis IOL was implanted into the eye, and subjects who have attended all postoperative examinations as per the protocol schedule. Exclusion criteria were previous intraocular and corneal surgery, pregnancy & lactation, traumatic and morgagnian cataract, pseudoexfoliation, concurrent participation in another drug or device study, chronic uveitis, patients receiving chloroquine or hydroxychloroquine treatment, corneal dystrophy or endothelial insufficiency, microphthalmia, axial length < 21 mm and > 24 mm, active ocular diseases (active diabetic retinopathy, uncontrolled glaucoma) and patients receiving α1 blockers. Only one eye of each patient was evaluated within the scope of the study. Follow-up data for all patients included in the study were recorded in the case report form (CRF) according to the clinical follow-up schedule, which consisted of 1 day, 7 days, 30 days, and 180 days.

A total of 6 visit data were considered in the study, out of which four follow-up visits were conducted on days 1, 7, 30, and 180 after the completion of cataract surgery. Clinical visits include preoperative, intraoperative, and postoperative visits at day 1, 7 days ± 2 days, 30 days ± 7 days, and 180 days ± 15 days.

### Study groups

The study was planned to be a two-arm performance goal-based endpoint-driven study. The primary endpoints were IOL tilt and IOL decentration. The secondary endpoints were UCVA and BCVA (time frame: preoperative visit, 1 day, 1 week, 30 and 180 days follow-up), contrast sensitivity (time frame: 30 and 180 days follow-up), manifest refraction (time frame: preoperative visit, 1 day, 1 week, 30 and 180 days follow-up), intraocular pressure (IOP): (time frame: pre-operative visit, 1 day, 1 week, 30 and 180 days follow-up) and PCO (time frame: 180 days follow-up).

Adverse events were summarized using the System Organ Classification (SOC) and reported in terms of severity, duration, onset time, and relationship to the Investigational Products, with appropriate action taken. The incidence of adverse events was summarized and compared across the treatment arms. In addition, the incidence of treatment-emergent adverse events, categorized by severity and relationship, was summarized and compared across the treatment arms. Serious adverse events were summarized separately.

### Handling of dropouts or missing data

All subjects who discontinued the study were excluded from efficacy evaluation for statistical analysis. All subjects who had all study data were included for efficacy evaluation, except subjects 007, 015, 016, 026, 041, 042, 043, 044, 045, 046, 047, 048, 049, 050, 052, 073, 078, 081, and 084, who did not have the follow-up data.

### Surgery

All surgeries were performed by the same surgeon (Y.S.G.) with topical anestesia. After a precise corneal incision (2.8 mm) and one side port incision, an anterior chamber was formed. A continuous curvilinear capsulorhexis of approximately 5 to 5.5 mm was performed. After phacoemulsification and coaxial I/A, a hydrophobic monofocal IOL-Optiflex Genesis or Eyecryl Plus ASHFY600 was implanted.

### IOL tilt and IOL decentration calculations

**Fig. 1:**
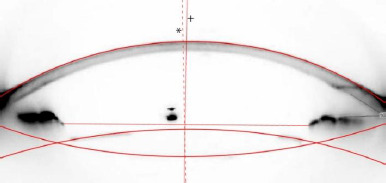
The visual and lens axis. The dashed line with an asterisk shows the intraocular lens axis, and the plus sign shows the pupillary axis

Following mydriasis, cross-sectional images of the implanted IOL were taken using Pentacam HR (Oculus Optikgerate GmbH, Wetzlar, Germany). Images at the 90^0^ and 180^0^ meridians were used for IOL position calculations. IOL position calculations were performed based on the technique described by de Castro et al. [11]. Calculations and measurements were made by the same person (S.O.), who was blinded to the patient group, to enhance the reliability and reproducibility. Adobe Photoshop (version CS5; Adobe) software was used for image processing measurements. Contrasts of the images were adjusted to acquire distinct IOL edges. Possible deformation in the cross-sectional images was corrected based on Castro et al.’s descriptions [11]. Perfectly fitted circumferences were placed at the anterior and posterior edges of the IOL, and the line passing through the intersection of the circumferences indicated the lens axis, and the midpoint of that line indicated the estimated center of the IOL. The midpoint of the line that connects the opposite edges of the pupil indicates the estimated center of the pupil. The line passing through the perfectly fitted circumference of the anterior curvature of the cornea and the center of the pupil indicated the pupillary axis (**Fig. 1**). Intraocular lens decentration was calculated as the distance between the pupillary axis and the center of the IOL. The angle of IOL tilt was calculated from the angle between the pupillary axis and the IOL axis.

### Statistical analysis

The sample size was calculated based on the primary endpoint, IOL tilt, of the study. At least 74 eyes were required to detect a difference of 1.28 degrees in IOL tilt compared to the comparator to achieve 80% power with a 5% level of significance. Considering the dropout rate of up to 20%, a total of 94 eyes were required in the study. Unfortunately, because of the acute respiratory syndrome coronavirus disease (COVID-19) outbreak in the world between 2019 and 2022, 19 patients did not have follow-up data. In the study, 85 eyes were enrolled, and 66 of them were considered for statistical analysis. IOL tilt and IOL decentration are summarized using descriptive statistics (minimum, maximum, median, mean, and standard deviation). The quantitative secondary endpoints were summarized using descriptive statistics (minimum, maximum, median, mean, and standard deviation). The change from baseline was calculated and summarized using descriptive statistics (minimum, maximum, median, mean, and standard deviation). The data for UCVA and BCVA were recorded using a Snellen chart at a distance of 4 m on the preoperative visit, as well as 1 day, 1 week, 30 days, and 180 days after surgery, and were compared. The results were analyzed in LogMAR. The change from baseline was also compared using a t-test, and the corresponding p-values were reported. The endpoints were plotted using error bars and/or histograms. The statistical analysis was performed using an appropriate SAS® Version 9.2 or higher.

## Results

**Fig. 2:**
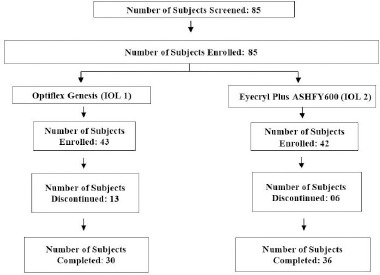
Subject disposition - all screened subjects

A total of 85 subjects’ medical records were screened, out of which 43 subjects’ medical records were enrolled in the IOL 1 (Optiflex Genesis) arm and 42 subjects’ medical records were enrolled in the IOL 2 (Eyecryl Plus ASHFY600) arm. Among all enrolled subjects, 30 (69.77%) and 36 (85.71%) subjects have completed the study in the IOL 1 and IOL 2 arms, respectively (**Fig. 2**). Moreover, 13 (30.23%) and 6 (14.29%) subjects discontinued the study due to loss to follow-up in the IOL 1 and IOL 2 arms, respectively. All discontinued subjects were excluded from the analysis population.

The demographic characteristics of the study participants are shown in **Table 1**.

**Table 1:** Summary of subject demographics and characteristics – analysis population

Demographics Characteristics	Optiflex Genesis (N=30)	Eyecryl Plus ASHFY600 (N=36)
**Age (Years)**		
N	30	36
Mean ± SD	64.20 ± 10.14	62.08 ± 9.80
Median	66	64
Min, Max	45,81	42,81
**Sex, n (%)**		
Male	23 (76.67)	22 (61.11)
Female	7 (23.33)	14 (38.89)

Note: Abbreviation: N = number of subjects in specified treatment; n = number of subjects in the specified category; Percentages are based on the number of subjects in the specified treatment.

### Axial Length Values

The mean axial length of the IOL 1 and IOL 2 arms was 23.07 ± 0.38 (21.92–23.34) mm and 23.06 ± 0.49 (21.86–23.49) mm, respectively. There were no statistically significant differences (p>0.05) between the IOL 1 arm and the IOL 2 arm.

### Primary efficacy endpoint results

The data on tilt and decentration, measured in both vertical and horizontal positions using Pentacam, were compared 180 days after surgery. The mean (±SD) of vertical IOL tilt at postoperative 180 days (Visit 6) was 1.41° (±0.72) and 1.25° (±0.85) for IOL 1 arm and IOL 2 arm, respectively (p>0.05). The mean (±SD) of horizontal IOL tilt at postoperative 180 days (Visit 6) was 1.29° (±0.92) and 0.94° (±0.83) for IOL 1 arm and IOL 2 arm, respectively (p>0.05).

The mean (±SD) of vertical IOL decentration at postoperative 180 days (Visit 6) was 0.25 (±0.09) mm and 0.26 (±0.14) mm for IOL 1 arm and IOL 2 arm, respectively (p>0.05). The mean (±SD) of horizontal IOL decentration at postoperative 180-day visit (Visit 6) was 0.21 (±0.10) mm and 0.23 (±0.12) mm for IOL 1 and IOL 2 arms, respectively (p>0.05).

### Secondary endpoint results

**Fig. 3:**
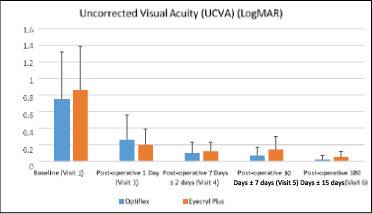
Summary of Uncorrected Visual Acuity (UCVA) between IOL 1 (Optiflex Genesis) and IOL 2 (Eyecryl Plus ASHFY600) for all visits

For the analysis population, the mean (±SD) of UCVA in the IOL 1 arm was 0.75 (±0.57) LogMAR at baseline. The changes in UCVA in the IOL 1 arm from baseline were -0.50 (±0.52), -0.65 (±0.52), -0.68 (±0.54), and -0.73 (±0.56) LogMAR at day 1, day 7, day 30, and day 180, respectively. The mean (±SD) of UCVA in the IOL 2 arm was 0.86 (±0.53) LogMAR at baseline. The changes in UCVA in the IOL 2 arm from baseline were -0.67 (±0.58), -0.74 (±0.54), -0.73 (±0.52), and -0.81 (±0.53) LogMAR at day 1, day 7, day 30, and day 180, respectively (**Fig. 3**). Within-group analysis, a significant difference (p<0.05) was observed at all visits from baseline for the IOL 1 arm and the IOL 2 arm. In the between-group analysis, there were no statistically significant differences (p>0.05) at all visits in UCVA between the IOL 1 arm and the IOL 2 arm.

**Fig. 4:**
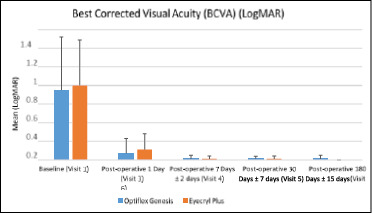
Summary of Best Corrected Visual Acuity (BCVA) between IOL 1 (Optiflex Genesis) and IOL 2 (Eyecryl Plus ASHFY600) for all visits

The mean (±SD) of BCVA in the IOL 1 arm was 0.75 (±0.57) LogMAR at baseline. The changes in BCVA in the IOL 1 arm from baseline were -0.68 (±0.53), -0.74 (±0.54), -0.74 (±0.57), and -0.74 (±0.57) LogMAR at day 1, day 7, day 30, and day 180, respectively. The mean (±SD) of BCVA in the IOL 2 arm was 0.80 (±0.49) LogMAR at baseline. The changes in BCVA in the IOL 2 arm from baseline were -0.69 (±0.53), -0.79 (±0.50), -0.79 (±0.49), and -0.80 (±0.49) LogMAR at day 1, day 7, day 30, and day 180, respectively (**Fig. 4**). Within-group analysis, a significant difference (p<0.05) was observed at all visits from baseline for the IOL 1 and IOL 2 arms. In the between-group analysis, there were no statistically significant differences (p > 0.05) at any visit in BCVA between the IOL 1 and IOL 2 arms.

**Fig. 5:**
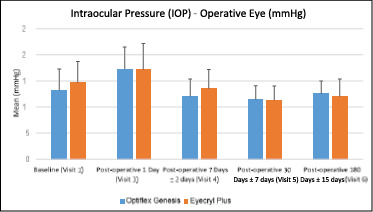
Summary of Intraocular Pressure (IOP) - Operative Eye between IOL 1 (Optiflex Genesis) and IOL 2 (Eyecryl Plus ASHFY600) for all visits

The intraocular pressure (IOP) data measured by tonometer at the preoperative visit, 1 day, 1 week, 30 days, and 180 days follow-up after surgery were compared. The mean (±SD) of IOP-operative eyes in the IOL 1 arm was 13.20 (±4.08) mmHg at baseline. The changes in IOP - operative eye in IOL 1 arm from baseline were 4.07 (±4.93), -1.20 (±4.18), -1.77 (±3.41), and -0.67 (±4.58) mmHg on day 1, day 7, day 30, and day 180, respectively. The mean (±SD) of IOP-operative eyes in the IOL 2 arm was 14.67 (±4.07) mmHg at baseline. The changes in IOP - operative eye in IOL 2 arm from baseline were 2.47 (±5.96), -1.17 (±4.81), -3.39 (±3.46), and -2.69 (±3.89) mmHg at day 1, day 7, day 30, and day 180, respectively. Within-group analysis revealed a significant difference (p < 0.05) on days 1 and 30, whereas no significant difference (p < 0.05) was observed on days 7 and 180 from baseline for IOL 1. Within-group analysis, an important difference (p<0.05) was observed on day 1, day 30, and day 180, whereas a significant difference (p<0.05) was not observed at day 7 from baseline for the IOL 2 arms. In the between-group analysis, there were no statistically significant differences (p>0.05) at all visits in IOP - operative eyes between IOL 1 and IOL 2 arms (**Fig. 5**).

The data of manifest refraction performed by auto refraction (automated) and manual procedures (standard) on preoperative visits, 1 day, 1 week, 30, and 180 days follow-up after surgery were compared.

For the analysis population, the mean (±SD) of manifest refraction (Sphere) in the IOL 1 arm was -1.95 (±2.47) diopters at baseline. The mean (±SD) of manifest refraction (Sphere) in the IOL 1 arm was 0.37 (±0.62), 0.23 (±0.63), 0.25 (±0.36), and 0.50 (±0.59) diopters at day 1, day 7, day 30, and day 180, respectively.

The mean (±SD) of manifest refraction (Sphere) in the IOL 2 arm was -2.25 (±0.50) diopters at baseline. The mean (±SD) of manifest refraction (Sphere) in the IOL 2 arm was 0.24 (±0.66), 0.31 (±0.59), 0.17 (±0.43), and 0.12 (±0.47) diopters at day 1, day 7, day 30, and day 180, respectively. In the between-group analysis, there were no statistically significant differences (p>0.05) on day 1, day 7, and day 30 in manifest refraction (Sphere) between IOL 1 and IOL 2 arms. There was a statistically significant difference (p<0.05) at day 180 in manifest refraction (Sphere) between IOL 1 and IOL 2 arms (**Table 2**).

**Table 2:** Summary of secondary efficacy endpoint [manifest refraction (sphere)]

Manifest Refraction (Sphere) (Diopters)			
Visit	Statistics	Optiflex Genesis	Eyecryl Plus ASHFY600
Baseline (visit 1)	N	30	36
	Mean ± SD	-1.95 ± 2.47 D	-2.25 ± 0.50 D
	Median	-1.95	-2.00
	Min, Max	-3.70, -0.20	-3.00, -2.00
	p-value	0.8047	
Post-operative 1 day (visit 3)	N	30	36
	Mean ± SD	0.37 ± 0.62 D	0.24 ± 0.66 D
	Median	0.00	0.00
	Min, Max	-1.00, 1.50	-1.00, 2.75
	p-value	0.4562	
Post-operative 7 days (Visit 4)	N	30	36
	Mean ± SD	0.23 ± 0.63 D	0.31 ± 0.59 D
	Median	0.00	0.25
	Min, Max	-1.20, 1.50	-1.00, 2.25
	p-value	0.6120	
Post-operative 30 days (visit 5)	N	30	36
	Mean ± SD	0.25 ± 0.36 D	0.17 ± 0.43 D
	Median	0.13	0.00
	Min, Max	-0.50, 1.00	-0.70, 1.00
	p-value	0.4265	
Post-operative 180 days (visit 6)	N	30	36
	Mean ± SD	0.50 ± 0.59 D	0.12 ± 0.47 D
	Median	0.38	0.00
	Min, Max	0.00, 2.00	-1.20, 1.00

Note: Abbreviation: N = number of subjects; If the P-value is less than 0.05, the conclusion is that the mean difference between the groups (Optiflex Genesis and Eyecryl Plus ASHFY600) is statistically significant.

The data on contrast sensitivity, measured using the Pelli-Robson Contrast Sensitivity Chart, were compared at 30 days and 180 days after surgery. The mean (±SD) of contrast sensitivity score in the IOL 1 arms was 0.34 (±0.13) and 0.36 (±0.11) log units at day 30 and day 180, respectively. The mean (±SD) of contrast sensitivity score in the IOL 2 arms was 0.37 (±0.10) and 0.37 (±0.08) log units at day 30 and day 180, respectively. In the between-group analysis, there were no statistically significant differences (p>0.05) at day 30 and day 180 (Visit 6) in contrast sensitivity between IOL 1 and IOL 2 arms (**Table 3**).

**Table 3:** Summary of secondary efficacy endpoint contrast sensitivity

Contrast Sensitivity (Mesopic) (log unit)			
Visit	Statistics	Optiflex Genesis	Eyecryl Plus ASHFY600
Post-operative 30 days (visit 5)			
	N	30	36
	Mean ± SD	0.34 ± 0.13 log unit	0.37 ± 0.10 log unit
	Median	0.41 log unit	0.41 log unit
	Min, Max	-0.11, 0.41	0.05, 0.50
	p-value	0.2287	
Post-operative 180 days (visit 6)			
	N	30	36
	Mean ± SD	0.36 ± 0.11 log unit	0.37 ± 0.08 log unit
	Median	0.41 log unit	0.41 log unit
	Min, Max	0.05, 0.50	0.18, 0.50
	p-value	0.6644	

Note: Abbreviation: N = number of subjects; If the P-value is less than 0.05, the conclusion is that the mean difference between the groups (Optiflex Genesis and Eyecryl Plus ASHFY600) is statistically significant.

For the analysis population, the mean (±SD) PCO score in the IOL 1 arm was 0.03 (±0.18) points at day 30 and 0.17 (±0.38) points at day 180, respectively. The mean (±SD) of PCO score in the IOL 2 arms was 0.03 (±0.17) points and 0.06 (±0.23) points at day 30 and day 180, respectively. In the between-group analysis, there were no statistically significant differences (p>0.05) at day 30 and day 180 (Visit 6) in PCO scores between IOL 1 and IOL 2 arms.

The patients were examined during all visits, whether they presented with treatment-emergent adverse events (TEAEs), adverse drug reactions (ADRs), serious adverse events (SAEs), or any other complications. There were no TEAEs, ADRs, or any other complications reported or observed with the treatment. No deaths, SAEs, or any significant adverse events occurred in this study. There were no TEAEs observed or reported during the study.

## Discussion

This study aimed to evaluate IOL tilt and decentration after Optiflex Genesis (Biotech Vision Care, India) (Group 1) and Eyecryl Plus ASHFY600 (Biotech Vision Care, India) (Group 2) hydrophobic aspheric monofocal IOL implantation and to evaluate its visual outcomes, safety, and complications. The data of the two groups were then compared. There were no statistically significant differences in vertical (1.41 ± 0.72 degrees vs. 1.25 ± 0.85 degrees, p=0.4202) and horizontal (1.29 ± 0.92 degrees vs. 0.94 ± 0.83 degrees, p=0.1147) IOL tilt results. Moreover, there was no significant difference in horizontal decentration (0.21 ± 0.10 mm vs. 0.23 ± 0.12 mm, p = 0.6400) and vertical decentration (0.25 ± 0.09 mm vs. 0.26 ± 0.14 mm, p = 0.5695) between groups at 6 months postoperatively.

Tilt and decentration of IOL may occur secondary to complicated cataract surgery or following uneventful phacoemulsification. Although up to 2 to 3° tilt and 0.2 to 0.3 mm decentration are common and clinically unnoticed for any IOL design, a larger extent of tilt and decentration harms the optical performance and subsequently, the patient’s satisfaction [11]. Röggla et al. assessed decentration and tilt of a new hydrophobic acrylic IOL Nanex (NC1-SP; HOYA Surgical Optics) [12]. They reported that the median IOL rotation of all eyes from the end of surgery to 6 months was 1.9 degrees [12]. Wandelstein and colleagues evaluated the rotational stability of a new monofocal IOL with a 7.0 mm optic and frame haptics [13]. IOL rotation was within 5 degrees in 85% of their study population at the 4-month follow-up [13]. In another study, Schartmüller and colleagues evaluated decentration and tilt of the Rayner RAO800C single-piece hydrophobic acrylic IOL [14]. Horizontal and vertical decentration at 4 months were -0.09 ± 0.14 and 0.09 ± 0.14 mm, respectively [14]. IOL vertical-horizontal tilt and decentration found in the current study are within the limits reported in the literature. The study results were consistent with those of the published study [14].

IOL tilt and decentration could be secondary to various etiologies. The extent and direction of preoperative crystalline lens tilt have been demonstrated to be strongly correlated with postoperative tilt [6,9]. Asymmetrical IOL fixation (partly in the bag and partly in the sulcus) and capsular tear during capsulorhexis were reported as some causative factors for IOL tilt and decentration [6,15]. In this study, none of the patients developed intraoperative complications. Capsular fibrosis, also known as asymmetric capsular fibrosis, may also trigger IOL tilt and decentration. According to the current literature, adhesion between the capsular bag and hydrophobic IOLs makes hydrophobic IOLs more resistant to capsular fibrosis [6,15]. In the present study, no patient developed prominent capsular fibrosis. The shape and diameter of the capsulorhexis are other factors influencing IOL position, and symmetric, small, and/or large capsulorhexis diameters have a significant impact on IOL position [16,17]. In our study, all surgeries were performed by the same surgeon (Y.S.G.) to prevent bias and distortion in the IOL position data.

When the pre- and postoperative UCVA and BCVA values were compared, a significant increase in visual acuity was observed in both the Optiflex Genesis and Eyecryl Plus ASHFY600 groups (p < 0.001 for both). In the between-group analysis, there were no significant differences (p > 0.05) at any visit in UCVA and BCVA between Optiflex Genesis and Eyecryl Plus ASHFY600.

IOP increased slightly in both groups on the first postoperative day. At one month postoperatively, the values of both groups had returned to their baselines. Early postoperative IOP elevation following cataract surgery is a frequent adverse event and might represent 88% of early postoperative complications [18]. The risk factors for IOP elevation following phacoemulsification surgery include residual viscoelastic material, glaucoma, and pseudoexfoliation syndrome [18]. We believe that the early postoperative IOP elevation observed in the current study after phacoemulsification surgery is due to the residual viscoelastic material.

Biber et al. reported a PCO development rate of 28% in the monofocal spherical group and 14.7% in the monofocal aspheric group [19]. PCO on Optiflex Genesis was 0.17 ± 0.38 points at 6 months, while on Eyecryl Plus ASHFY600, PCO was 0.06 ± 0.23 points at 6 months. In this study, PCO was graded as mild, moderate, or severe. According to our average score, the development of PCO was mild at the 6-month examination. There was no significant difference between the Optiflex Genesis and Eyecryl Plus ASHFY600 groups.

Limitations of the study include its retrospective nature and small sample size. Due to the COVID-19 outbreak worldwide between 2019 and 2022, many patients lacked follow-up data. A further limitation was that we were unable to assess higher-order aberrations. Hence, this study demonstrated that Optiflex Genesis and Eyecryl Plus ASHFY600 are comparable, presenting good clinical effectiveness and safety for patients during the evaluated period.

## Conclusion

This study aimed to evaluate IOL tilt and decentration after the implantation of Optiflex Genesis and Eyecryl Plus ASHFY600 hydrophobic aspheric monofocal IOLs. IOL tilt up to 2-3 degrees and decentration of 0.2-0.3 mm are common and not clinically significant. Larger amounts of decentration and tilt deteriorate the optical performance of the IOLs, subsequently affecting the patients’ vision. Optiflex Genesis and Eyecryl Plus ASHFY600 IOL tilt and decentration found in the current study are within the limits reported in the literature.
